# Endemic Yucatan Peninsula Plants with Pesticidal Potential: Herbarium-Based Literature Review

**DOI:** 10.3390/plants13243583

**Published:** 2024-12-22

**Authors:** Nancy Alonso-Hernández, Carlos Granados-Echegoyen, Baldomero H. Zárate-Nicolás, Demian Hinojosa-Garro, Esperanza Loera-Alvarado, Nadia Landero-Valenzuela, Beatriz Quiroz-González, Fidel Diego-Nava, Salvador Ordaz-Silva, Imelda Virginia López-Sánchez, Laura Dennisse Carrazco-Peña

**Affiliations:** 1 Instituto Politécnico Nacional (IPN), Centro Interdisciplinario de Investigación para el Desarrollo Integral Regional (CIIDIR), Campus Oaxaca, Santa Cruz Xoxocotlán 71230, Oaxaca, Mexico; alonsoh_nancy@hotmail.com (N.A.-H.); bquiroz@ipn.mx (B.Q.-G.); fdiego@ipn.mx (F.D.-N.); 2 CONAHCYT-Instituto Politécnico Nacional, CIIDIR Unidad Oaxaca, Santa Cruz Xoxocotlán 71230, Oaxaca, Mexico; 3 Centro de Estudios en Desarrollo Sustentable y Aprovechamiento de la Vida Silvestre (CEDESU), Universidad Autónoma de Campeche, San Francisco de Campeche 24079, Campeche, Mexico; dhinojos@uacam.mx; 4 CONAHCYT-Universidad Autónoma Chapingo, Centro Regional Universitario Centro Occidente (CRUCO), Morelia 58170, Michoacán, Mexico; loera6@hotmail.com; 5 Department of Horticulture, Universidad Autónoma Agraria Antonio Narro, Calzada Antonio Narro 25294, Coahuila, Mexico; nadia.landerov@uaaan.edu.mx; 6 Facultad de Ingeniería y Negocios San Quintín, Universidad Autónoma de Baja California, Mexicali 21100, Baja California, Mexico; salvador.ordaz.silva@uabc.edu.mx (S.O.-S.); lopezi13@uabc.edu.mx (I.V.L.-S.); laura.carrazco@uabc.edu.mx (L.D.C.-P.)

**Keywords:** endemic plants, pest control, plant-derived natural products, Mexico

## Abstract

Agricultural pests present a significant challenge to humanity, often managed through synthetic chemicals that, when misused, can cause irreversible harm to both the environment and human health. This study focuses on endemic plants from the Yucatán Peninsula in Mexico, particularly from the state of Campeche, to identify their historical uses and propose an updated list of species with pesticide potential in the region. We systematically reviewed specimens from the Center for Sustainable Development and Wildlife Management (CEDESU) herbarium and local databases. Of the 3084 specimens collected, 2524 (81.84%) were from Campeche. The collection encompasses 106 botanical families, 459 genera, and 747 species. The study identified 201 plant species from 48 taxonomic families that are endemic to the Yucatán Peninsula Biotic Province (YPBP), of which 123 species are exclusive to the Mexican Yucatán Peninsula (MYP), representing 61.19% of the endemic species. Campeche contains 134 species (66.66%), distributed across 96 genera and 43 families. Notably, 46.26% of the species (62 species) belong to the Mexican region, with 8 species (12.90%) exclusive to Campeche. The research revealed that 27.90% of the families and 19.79% of the genera present in the state have been the subject of previous scientific studies regarding their use as pesticides. The most extensively studied families were Euphorbiaceae and Fabaceae. However, there is a notable lack of research on endemic plants from the Yucatán Peninsula, underscoring the need for increased attention to these species. The identified genera and families contain chemical compounds with activity against significant pests, demonstrating substantial potential for the development of natural pesticides.

## 1. Introduction

Agricultural pests are a significant global issue because of their role in spreading diseases and causing substantial losses on important crops. They also degrade the quality of affected products, making them challenging to market [1]. According to Shashidhar et al. [2], a pest is any harmful organism that inflicts economic damage on crops and impacts non-target organisms within agroecosystems. Pests disrupt global food production, affecting crops during growth and sometimes even during post-harvest storage [3]. Synthetic chemicals. Synthetic chemicals have long been used to control pests in important crops. However, studies have found that some synthetic chemicals products like abamectin, cypermethrin, endosulfan, and imidacloprid can cause irreversible damage [4]. As demonstrated by Zhang et al. [5] in their study using mouse models, certain products can adversely affect the reproductive health of the subjects examined. Similarly, Kalefetoglu [6] highlights that abamectin is a harmful pesticide with a range of cytotoxic and genotoxic effects on non-target organisms.

The widespread and indiscriminate use of these pesticides not only promotes insect resistance but also results in environmental contamination, posing significant risks to human health and ecological balance [7]. To address these issues, it is crucial to develop pest control strategies that adopt an ecological approach. This includes exploring botanical alternatives for pest management, such as plant extracts with solvents, insecticidal plant powders, and essential oils. These natural options can serve as repellents, anti-feedants, insecticides, fungicides, herbicides, and nematicides [8].

The ethnobotanical use of natural products offers a viable and sustainable alternative for insect pest control due to the effectiveness of the secondary chemical compounds they contain. These compounds, such as monoterpenoids, sesquiterpenoids, phenylpropanoids, and alkaloids, are responsible for their insecticidal properties [9,10,11]. Plants have evolved over millions of years to develop defense mechanisms against insect attacks, including repellency and insecticidal actions [12].

Many plant species produce secondary metabolites with biological activity, which are extracted from roots, seeds, leaves, or fruits [13,14]. These extracts have led to the development of valuable products with insecticidal potential in adult and immature stages [15]. Additionally, these natural products help reduce the development of resistance to pests compared to synthetic insecticides [16]. Furthermore, many of these products adhere to international standards for environmentally friendly production [17].

Worldwide, there are 250,000 flowering plant species (Magnoliophyta), with about 22,351 species native to Mexico [18,19,20,21,22]. However, Mexico’s floral diversity is not yet documented, and a reliable total estimate remains elusive. According to Villaseñor [23], reports indicate that Mexico may have the highest level of endemism in the Americas and is recognized as a ‘megadiverse’ country, ranking among the top five in terms of floristic richness [24]. A recent study identifies 53 orders, 247 families, 2685 genera, and 21,841 species of flowering plants in Mexico, with 11,001 species being endemic [22].

Mexico consists of 32 states and is divided into eight regions, with the Southeast Region being one of them. This region includes the Yucatan Peninsula (MYP), covering the states of Campeche, Quintana Roo, and Yucatan, with a total area of 166,445.49 km^2^. Despite its size, the Yucatan Peninsula remains one of Mexico’s least explored and studied regions. Known as the Yucatan Peninsula Biotic Province (YPBP), it extends into the northern parts of Belize and Guatemala. Approximately 2327 plant species used for ethnobotanical purposes are found in the Yucatan Peninsula, distributed across 956 genera and 161 families. Among the families with the highest species richness are Fabaceae, Poaceae, Asteraceae, Orchidaceae, and Euphorbiaceae. According to Fernández et al. [25], the region’s total flora includes 99 endemic species, accounting for 4.27% [26]. While the Peninsula does not have high species diversity, it is of significant interest from a floristic and biogeographical perspective. This interest arises from the combination of elements from Central America, the Caribbean Sea Basin, and southern Mexico, mixed with endemic species, creating a unique flora [27,28]. The Yucatan Scientific Research Centre (CICY) from Mexico states that around 30% of the vascular plants in the MYP (648 species) have known medicinal uses [29]. According to the study by Méndez et al. [30], a total of 565 species across 370 genera and 107 families have been documented. However, most studies have focused on developing new drugs rather than exploring the potential of these plants for pest control in important crops.

To date, most research on plant-derived products has concentrated on their medicinal uses for antibacterial [31,32,33], antifungal [34,35,36], amebicidal [37], anti-inflammatory [38], sedative [39,40], spasmolytic [41], anti-arthritis [42], and antioxidant properties [43]. However, there is a lack of information regarding the efficacy of these plants in controlling arthropods, microorganisms, and weeds that are considered pests.

This review aims to assess the current status of endemic plants in the Yucatan Peninsula, with a particular focus on those in the state of Campeche that have been the least studied. It seeks to identify endemic plants utilized in agriculture and propose an updated list of species with potential pesticidal properties. The review will include species from the scientific collection of the herbarium at the Center for Studies in Sustainable Development and Wildlife Utilization (CEDESU) at the Autonomous University of Campeche (UACAM) and specialized literature. By addressing this knowledge gap, the review aspires to provide new insights into the pesticidal potential of endemic plants in the region. We hope that by highlighting the potential of these plants in southeastern Mexico, we can foster connections with various national and international institutions, thereby enabling the development of academic and research projects focused on discovering natural chemical alternatives to pesticides, reducing reliance on synthetic agrochemicals.

## 2. Results

**Figure 1:**
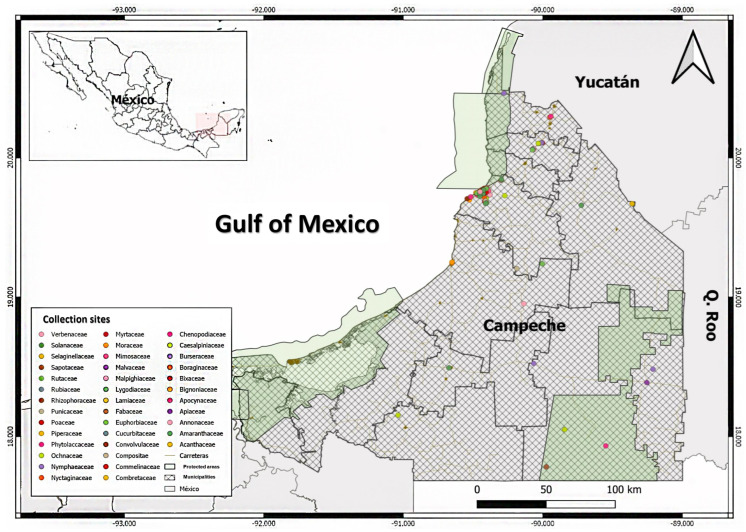
Distribution of botanical families among the specimens in the Herbarium of the Scientific Collection at CEDESU-UACAM, highlighting the proportion of species collected from the state of Campeche.

The review and organization of the material in the CEDESU-UACAM Herbarium revealed 3084 plant specimens collected from the states of Campeche, Chiapas, Oaxaca, and Veracruz. Among these specimens, 81.84% (2524) are from Campeche (Figure 1). The Campeche collection includes specimens from 106 botanical families, spread across 459 genera and 747 species. Notably, 83 species among these have yet to be identified by researchers.

Based on a review of the local literature, scientific databases, herbarium collections, and National Commission for the Knowledge and Use of Biodiversity of Mexico CONABIO’s website, we found that endemic species from each state in the Yucatan Peninsula (MYP)—which includes Campeche, Quintana Roo, and Yucatan—also exhibit endemism with other areas within the Yucatan Peninsula Biotic Province (YPBP). The YPBP extends to Guatemala and Belize as well. Within the YPBP, we identified 48 taxonomic families and 201 plant species from 135 endemic genera, with 123 species being exclusive to the MYP. This represents 61.19% of the species endemic to the Mexican region (Table 1). The compilation of a floristic list of YPBP endemic species found in Campeche revealed that 66.66% (134 species) of these species are present in the state. The distribution of these species spans across 96 genera and 43 botanical families. Among these, 46.26% (62 species) are exclusive to the Mexican region (MYP), and 12.90% (8 species) are exclusive to Campeche.

In the review of species occurring in the state of Campeche, it was found that 27.90% (n = 12) of the taxonomic families are reported in at least one study on pest control. Among the genera present in the state (n = 96), 19.79% (n = 19) have been investigated in previous studies. For species (n = 134), 17.16% (n = 23) have been mentioned in some form of study (Table 2).

Among the endemic species of previous studies, the most represented families were Euphorbiaceae and Fabaceae. The Euphorbiaceae family includes the following species: *Acalypha gaumeri, Cnidoscolus souzae, Croton arboreus, Croton chichenensis,* and *Jatropha gaumeri.* The Fabaceae family is represented by *Gliricidia maculata, Havardia albicans, Lonchocarpus castilloi, Lonchocarpus xuul, Lonchocarpus yucatanensis, Platymiscium yucatanum,* and *Senegalia gaumeri* (Table 3).

## 3. Discussion

The search for new biological resources that can be used, to ensure safety and satisfaction for humans, is crucial today. In this context, the wild flora of the Yucatan Peninsula, combined with the ethnobotanical knowledge of Mayan culture regarding its medicinal and gastronomic uses, represents a valuable resource for bio-prospecting.

A key aspect of bio-prospecting is the variability in bioactive compound content within plants of the same family or genus, which can depend on factors such as developmental and handling conditions. For example, promising results have been obtained from the evaluation of plants of the genus *Cnidoscolus* for antimicrobial activity against *Escherichia coli* (Enterobacteriaceae) and *Pseudomonas aeruginosa* (Pseudomonadaceae). This suggests that *C. souzae*, an endemic species of the Yucatan Peninsula, may possess compounds with similar bioactive properties. Some note that members of the same botanical family and species within the same genus may exhibit similar characteristics and potential due to their common evolutionary ancestry. They share a genetic pool that enables them to synthesize similar chemical compounds. By occupying analogous ecological niches, these species develop traits and compounds that enhance their survival in comparable environments. Different species can produce similar metabolites because they often conserve the biosynthetic pathways for metabolite production within their taxonomic groups [71,72,73,74].

Moreover, studies have shown that the Cactaceae family is renowned for its high levels of triterpenes and sterols, compounds that possess the potential to control insects and bacteria [47]. For example, *Selenicereus grandiflorus*, a species from this family, may contain terpenoids with biological efficacy. Assessments conducted in entomological cages have shown that aqueous extracts of *Croton itzaeus* (stem) are also effective against *M. javanica*. Plants contain terpenoids, which are compounds with an isoprene-based structure bound to oxygen [75]. Terpenoids affect insects by slowing food passage through the gut and reducing digestibility by inhibiting the secretion of digestive enzymes such as proteases. This inhibition leads to physical weakening and impaired growth and development. Saponins, with a chemical structure similar to insect molting hormones, act as inhibitors or antagonists, disrupting the molting and metamorphosis processes [76].

Despite the rich biodiversity of the Yucatan Peninsula, nobody has reported the use of several endemic species, such as the genus *Justicia*, in pest control. However, researchers have conducted studies on other species within the same genus, such as *Justicia spicigera* (Acanthaceae), and have discovered that they possess flavonoids and tannins, which are secondary compounds known for their insecticidal properties [77,78]. *Jatropha curcas* seeds contain the compound Jatropherol-I, which has insecticidal properties [79]. This finding suggests that *Jatropha gaumeri* may also possess similar compounds. *Jatropha curcas* seed extracts have shown efficacy against the maize weevil *Sitophilus zeamais* (Coleoptera: Curculionidae) in stored grains, without affecting the germination of treated seeds [80]. In addition, *J. gossypifolia* leaf extract has shown toxicity to *Spodoptera litura* (Lepidoptera: Noctuoidea) larvae (24-h LC_50_, 6.56 mgmL^−1^) [81].

Despite the existing knowledge, *J. gaumeri* remains an understudied species in insect control. However, studies have shown that species of the genus *Justicia* are a rich source of active biomolecules with diverse biological activities, including terpenes [82]. Species of the genus *Acacia* have shown insecticidal effects, as evidenced by aqueous, ethanolic, and acetonic extracts of the aerial parts of *Acacia modesta*, which affect adults of the mosquito *Culex pipiens* [83]. However, there has been no evaluation of the species *Acacia gentlei* in pest control.

In our study, we found a significant diversity of plant species from several botanical families that are effective in pest control. However, there are currently no reports of products derived from endemic species of Campeche and the Yucatan Peninsula (YMP) being used for pest insect management. This underscores an obvious need to continue the search for biomolecules with the potential to be integrated into ecological strategies within integrated pest management.

Several organisms produced natural products, which comprise organic molecules with complex chemical structures, and include both primary and secondary metabolites. Secondary metabolites, in particular, are small organic molecules that are not essential for the growth, development, or reproduction of the producing organism. Instead, they are synthesized at specific stages of the life cycle or under particular environmental conditions [84,85]. We can classify these compounds based on their composition, chemical structure, synthesis route, or solubility in different solvents. A simple classification based on chemical structure includes three major groups: phenolic compounds (such as coumarins, flavonoids, and tannins), nitrogen compounds (alkaloids), and carbon and hydrogen compounds (terpenoids) [86,87,88].

Plant-produced metabolites serve as defense mechanisms and exhibit a broad spectrum of activity, affecting insects at cellular, tissue, or general organism levels [86]. These metabolites disrupt cellular and physiological processes responsible for homeostasis, leading to insecticidal effects such as inhibition of feeding, alterations in development, reduced fecundity, deformations in successive generations, interference with vital enzyme activity, alterations in the nervous system, blockage of metabolic pathways, behavioral impairment, and reduced insect populations [85,89,90,91,92,93,94,95,96].

For instance, alkaloids are prevalent and active compounds, with over 12,000 variants described to date [86]. Studies have shown that alkaloids exhibit a wide range of biological activities by interfering with various physiological mechanisms in insects. When alkaloids enter an insect, they trigger the production of reactive oxygen species (ROS), such as oxygen ions, free radicals, and peroxides. This increase in ROS leads to oxidative stress, which changes the mitochondrial membrane potential and causes cell death. Alkaloids prompt the opening of cell membrane channels, elevating calcium ion levels within the cell and leading to apoptosis [85,97,98].

Alkaloids also affect the hormonal balance of insects, during metamorphosis. They can disrupt the function of the prothoracicotropic hormone (PTTH), which activates the prothoracic glands to synthesize ecdysone (the molting hormone) and juvenile hormone. Disruption in the production of these hormones can cause larvae to remain in the larval stage for an extended period or, sometimes, reach the pupal stage but emerge with malformations such as deformed wings and reduced fecundity [99,100]. Alkaloids also have potent effects on the nervous system of insects. They mimic acetylcholine, an essential neurotransmitter, by binding to acetylcholine receptors on the cell membrane. This interaction alters membrane permeability, leading to spasmodic contractions, convulsions, and death of the insect [101]. Alkaloids have neurotoxic effects that manifest as decreased locomotion, tremors in appendages and abdominal segments, and altered food intake. These effects can lead to reproductive disturbances, such as reduced fecundity in females, inhibited sexual maturity, and decreased hatching rates [102].

Plants also produce flavonoids, which are compounds derived from the shikimate and acetyl coenzyme A pathways. Flavonoids represent one of the most diverse chemical groups, with over 5000 compounds identified [75]. These compounds disrupt the detoxification system of insects by reducing the activity of glutathione-S-transferase and esterase enzymes and decreasing mitochondrial activity. This reduction affects ubiquinone oxidoreductase, an enzyme crucial for energy production, affecting feeding and movement processes [103]. Tannins are another class of complex phenolic compounds, classified into hydrolyzable and condensed tannins. Their primary effect on insects is to cause fatal midgut injury through oxidative stress induced by peroxides generated during tannin oxidation [104,105].

Researchers have attributed the biosynthesis of bicyclic aromatic compounds called coumarins to the shikimate pathway [106]. These compounds are further classified into four groups: hydroxycoumarins, furanocoumarins, pyranocoumarins, and glycosylated coumarins [107]. Coumarins conjugate with the enzymes transaminase and cytochrome P450, inhibiting the detoxification system of insects [108]. We can extract plant compounds using various methods, both conventional (such as maceration and boiling) and non-conventional (such as microwaving and ultrasound), among others. Essential oils derived from these plants exhibit a range of mechanisms of action. Their primary property is the ability to alter the lipid bilayer of cells. Some essential oils can have synergistic effects when combined with botanical insecticides, enhancing their efficacy up to sixfold. These oils may exhibit multiple effects, including direct toxicity, growth inhibition, repulsion, and alteration of insect behavior [109,110].

Endemic botanical species from Campeche and the Yucatan Peninsula, belonging to taxonomic families known for pest control, have considerable potential in this area. However, the future use of these species for agricultural pest management is uncertain. Most current research focuses on identifying and characterizing chemical compounds for pharmaceutical applications, rather than exploring their potential in pest control. This underutilization contrasts with the progress made in other regions, where endemic flora has been effectively harnessed for developing plant-based biopesticides.

India and China lead globally in this field, leveraging their biodiversity and traditional use of botanical extracts with insecticidal properties. The United States follows in third place, emphasizing the technological production and commercialization of biopesticides [111]. Examples of endemic species with insecticide properties include *Azadirachta indica* (Meliaceae) from India, valued for its natural insecticidal extracts [112]; *Sophora flavescens* (Fabaceae) from China, effective against insects and fungi [113]; and *Maclura pomifera* (Moraceae), an endemic U.S. species recognized for its insect-repellent properties [114].

In comparison, species endemic to the Yucatan Peninsula, such as *C. souzae* and *J. gaumeri*, remain largely unexplored despite the region’s rich ethnobotanical tradition. This highlights the need for increased research investment to identify and characterize the secondary metabolites of these plants and integrate them into ecological pest management strategies. The experiences of India and China could serve as valuable models for advancing the development and adoption of biopesticides from local botanical resources.

## 4. Materials and Methods

A comprehensive review of relevant research articles was conducted by querying two major academic databases, SCOPUS and Web of Science (WOS), over the past ten years. The objective was to identify studies that assessed the insecticidal activity and chemical composition of endemic plants.

Additionally, a review was carried out on the botanical material stored in the Herbarium and Scientific Collection of CEDESU-UACAM, along with the local literature by Carnevali et al. [115] and Valencia-Gutiérrez et al. [116]. The search terms used included ‘insecticidal activity’, ‘pesticide’, ‘endemic’, ‘chemical composition’, and ‘Mexico’, and these terms were applied to the abstract, title, and keyword fields in both English and Spanish.

The database of the National Commission for the Knowledge and Use of Biodiversity of Mexico (CONABIO, https://www.biodiversidad.gob.mx, accessed on 1 January 2024) was also consulted. Duplicates were removed from the citation list, and the abstracts and full texts of each manuscript were reviewed. This process resulted in an updated list of endemic plants from the Yucatan Peninsula (MYP) and the Yucatan Peninsula Biotic Province (YPBP), including those specific to the state of Campeche. The database searches and the literature review were finalized on 15 July 2024.

## 5. Conclusions

The literature review shows that there is a significant limitation in research on the endemic plants of the Yucatan Peninsula. This research gap emphasizes the urgent need to shift focus towards these unique species. The genera and taxonomic families found in this region have shown the potential to harbor chemical compounds that could act on important pests, showing that they could provide valuable opportunities for the development of new pest control strategies. Despite their potential, there is a scarcity of existing sources addressing extraction technologies, chemical analysis, and pest control related to these species. This highlights a significant knowledge gap and emphasizes the need for more in-depth studies in these areas. Further research into the extraction of bioactive compounds, their chemical characterization, and their efficacy in pest control could not only expand our understanding of the endemic plants of the Yucatan Peninsula but also contribute to the development of more sustainable and effective pest management methods.

## Figures and Tables

**Table 1:** Updated list of endemic plants in the Yucatan Peninsula Biotic Province (YPBP), along with their distribution in the Mexican states comprising the Yucatan Peninsula (MYP).

Family	Species	MYP	YPBP
Acanthaceae	*Carlowrightia yucatanensis* T. F. Daniel	Yucatan	
	*Holographis websteri* T. F. Daniel	Campeche, Quintana Roo, Yucatan	
	*Justicia campechiana* subsp. *campechiana* Standl.	Campeche, Quintana Roo, Yucatan	*
	*J. cubensis* Lundell	Quintana Roo	
	*J. dendropila* T. F. Daniel	Quintana Roo	
	*J. edgarcabrerae* T. F. Daniel, Carnevali & Tapia	Quintana Roo	
	*J. leucothamna* (Standl.) T. F. Daniel, Carnevali & Tapia	Campeche, Yucatan	
	*J. lundellii* Leonard	Campeche, Quintana Roo, Yucatan	
	*J. luzmariae* T. F. Daniel, Carnevali & Tapia	Campeche, Quintana Roo	*
	*Stenandrium nanum* (Standl.) T. F.Daniel	Quintana Roo, Yucatan	
	*S. subcordatum* Standl.	Campeche, Quintana Roo, Yucatan	*
Agavaceae	*Furcraea cahum* (Jacq.) Urb.	Campeche, Quintana Roo, Yucatan	
	*Manfreda paniculata* L. Hern., R. A. Orellana & Carnevali	Quintana Roo, Yucatan	
	*M. petskinil* R. A. Orellana, L. Hern. & Carnevali	Quintana Roo, Yucatan	
Amaryllidaceae	*Zephyranthes orellanae* Carnevali, Duno & J. L. Tapia	Yucatan	
Anacardiaceae	*Attilaea abalak* E. Martínez & Ramos	Campeche, Quintana Roo	*
Anthericaceae	*Echeandia campechiana* Cruden	Campeche	**
	*E. luteola* Cruden	Campeche, Quintana Roo, Yucatan	
Apocynaceae	*Dictyanthus aeneus* Woodson	Campeche, Yucatan	
	*D. yucatanensis* Standl.	Campeche, Quintana Roo, Yucatan	
	*Gonolobus stenanthus* (Standl.) Woodson	Campeche, Quintana Roo, Yucatan	*
	*Marsdenia calichicola* Carnevali & Juárez-Jaimes	Yucatan	
	*Matelea belizensis* (Lundell & Standl.) Woodson	Quintana Roo, Yucatan	
	*M. belizensis* (Lundell & Standl.) Woodson & Matelea campechiana (Standl.) Woodson	Quintana Roo	
	*M. campechiana* (Standl.) Woodson	Campeche, Quintana Roo, Yucatan	*
	*M. crassifolia* Woodson	Campeche, Quintana Roo, Yucatan	
	*M. gentlei* (Lundell & Standl.) Woodson	Campeche, Quintana Roo, Yucatan	*
	*M. pusilliflora* L. O. Williams	Campeche, Quintana Roo	*
	*M. stenosepala* Lundell	Campeche, Quintana Roo, Yucatan	
	*Metastelma yucatanense* W. D. Stevens	Quintana Roo, Yucatan	*
Araceae	*Xanthosoma yucatanense* Engl.	Quintana Roo, Yucatan	
Arecaceae	*Coccothrinax readii* H. J. Quero	Quintana Roo, Yucatan	
	*Gaussia maya* (O. F. Cook) H. J. Quero & Read	Campeche, Quintana Roo	*
	*Sabal gretherae* H. J. Quero	Quintana Roo	
Asteraceae	*Acmella filipes var. filipes* (Greenm.) R. K. Jansen	Campeche, Quintana Roo, Yucatan	
	*Ageratum munaense* R. M. King & H. Rob.	Yucatan	
	*Calea urticifolia var. yucatanensis* Wussow, Urbatsch & G. A. Sullivan	Yucatan	
	*Critonia campechensis* (B. L. Rob.) R. M. King & H. Rob.	Campeche, Quintana Roo, Yucatan	*
	*Critoniopsis colepis* (S. F. Blake) H. Rob.	Campeche, Quintana Roo, Yucatan	
	*Goldmanella sarmentosa* Greenm.	Campeche, Quintana Roo, Yucatan	*
	*Otopappus guatemalensis* (Urb.) R. L. Hartm. & Stuessy	Campeche, Quintana Roo, Yucatan	*
	*Parthenium schottii* Greenm.	Yucatan	
	*Plagiolophus millspaughii* Greenm.	Campeche, Yucatan	
	*Pluchea yucatanensis* G. L. Nesom	Campeche, Quintana Roo	
	*Zyzyxia lundellii* (H. Rob.) Strother	Campeche	*
	*Parthenium schottii* Greenm.	Yucatan	
Brassicaceae	*Cakile lanceolata* subsp. *alacranensis* (Millsp.) Rodman	Campeche, Quintana Roo, Yucatan	
Bromeliaceae	*Hechtia schottii* Baker	Campeche, Yucatan	
	*Hohenbergia mesoamericana* I. Ramírez, Carnevali & Cetzal	Quintana Roo	
	*Tillandsia dasyliriifolia* Baker	Campeche, Quintana Roo, Yucatan	*
	*T. maya* I. Ramírez & Carnevali	Yucatan	
	*T. may-patii I.Ramírez & Carnevali*	Quintana Roo	
	*T. pseudobaileyi* spp. *yucatanensis* I. Ramírez	Campeche, Quintana Roo	
	*Wittmackia mesoamericana* (I. Ramírez, Carnevali & Cetzal) Aguirre-Santoro	Quintana Roo	
Cactaceae	*Mammillaria gaumeri* (Britton & Rose) Orcutt	Quintana Roo, Yucatan	
	*Nopalea gaumeri* Britton & Rose	Campeche, Quintana Roo, Yucatan	
	*N. inaperta* Schott ex Griffiths	Campeche, Quintana Roo, Yucatan	
	*Pilosocereus gaumeri* (Britton & Rose) Backeb.	Quintana Roo, Yucatan	
	*Pterocereus gaumeri* (Britton & Rose) T.MacDoug. & Miranda	Yucatan	
	*Selenicereus grandiflorus* subsp. *donkelaarii* (Salm-Dyck) Ralf Bauer	Campeche, Quintana Roo, Yucatan	*
Campanulaceae	*Lobelia yucatana* E. Wimm.	Campeche, Yucatan	
Capparaceae	*Quadrella incana* subsp. *yucatensis* (Lundell) Iltis	Campeche, Quintana Roo, Yucatan	
	*Q. isthmensis* subsp. *Mexicana* Cornejo & Iltis	Campeche, Quintana Roo, Yucatan	*
	*Q. lindeniana* Cornejo & Iltis	Campeche, Quintana Roo, Yucatan	
	*Q. quintanarooensis* Iltis & Cornejo	Quintana Roo	
Celastraceae	*Wimmeria lundelliana* Carnevali, R. Duno, J. L. Tapia & I. Ramírez	Campeche, Quintana Roo	
	*W. obtusifolia* Standl.	Quintana Roo, Yucatan	
Convolvulaceae	*Cuscuta palustris* Yunck.	Yucatan	
	*C. yucatana* Yunck.	Yucatan	
	*Ipomea sororia* D. F. Austin & J. L. Tapia	Campeche, Yucatan	
	*I. steerei* (Standl.) L. O. Williams.	Campeche, Quintana Roo, Yucatan	*
Cyperaceae	*Fuirena stephani* Ramos & Diego	Campeche	**
Dioscoreaceae	*Dioscorea gaumeri* R. Knuth	Campeche, Quintana Roo, Yucatan	*
Ebenaceae	*Diospyros bumelioides* Standl.	Campeche, Quintana Roo	*
	*D. yucatanensis* subsp. *longipedicellata* (Lundell) Provance, I.García & A.C. Sanders.	Yucatan	
	*D. yucatanensis* subsp. *spectabilis* (Lundell) Provance, I. García & A.C. Sanders	Campeche, Quintana Roo, Yucatan	*
Erythroxylaceae	*Erythrosylum becquaertii* Standl.	Campeche, Quintana Roo, Yucatan	*
Euphorbiaceae	*Acalypha gaumeri* Pax & K. Hoffm.	Campeche, Quintana Roo, Yucatan	
	*Argythamnia tinctoria* Millsp.	Yucatan	
	*A. wheeleri.* J. W. Ingram	Yucatan	
	*Bernardia yucatanensis* Lundell	Campeche, Quintana Roo, Yucatan	*
	*Cnidoscolus souzae* Mc. Vaugh	Campeche, Quintana Roo, Yucatan	*
	*Croton ameliae* Lundell	Quintana Roo, Yucatan	
	*C. arboreus* Millsp.	Campeche, Quintana Roo, Yucatan	*
	*C. chichenensis* Lundell	Campeche, Quintana Roo, Yucatan	*
	*C. icche* Lundell	Campeche, Quintana Roo, Yucatan	
	*C. mayanus* B. L. León & H. F. M. Vester	Campeche, Quintana Roo, Yucatan	
	*C. millspaughii* Standl.	Quintana Roo, Yucatan	
	*C. peraeruginosus* Croizat	Campeche, Quintana Roo, Yucatan	
	*Croton* sp. Müll. Arg.	Campeche, Quintana Roo, Yucatan	
	*Dalechampia schottii var. schottii* Müll. Arg.	Campeche, Quintana Roo, Yucatan	*
	*D. schottii var. Trifoliata* Greenm.	Campeche, Quintana Roo, Yucatan	
	*Enriquebeltrania crenatifolia* (Miranda) Rzed.	Campeche, Quintana Roo, Yucatan	
	*Euphorbia barbicarina* (Millsp.) Standl.	Campeche, Quintana Roo, Yucatan	
	*E. floresii* Standl.	Yucatan	
	*E. gaumeri* Millsp.	Quintana Roo, Yucatan	
	*E. xbacensis* Millsp.	Campeche, Yucatan	*
	*Jatropha gaumeri* Greenm.	Campeche, Quintana Roo, Yucatan	*
Fabaceae	*Acacia x cedilloi* L. Rico	Quintana Roo	
	*A. gentlei* Standl.	Campeche, Quintana Roo	*
	*Bauhinia erythrocalyx* Wunderlin	Campeche, Quintana Roo	*
	*Calliandra mayana* H. M. Hern.	Campeche	**
	*Dalea scandens* (Houst. Ex Mill.) R. T. Clausen	Yucatan	
	*Diphysa paucifoliolata* R. Antonio y M. Sousa	Campeche, Quintana Roo	*
	*D. yucatanensis* Hanan-Alipi & M. Sousa	Campeche, Quintana Roo, Yucatan	*
	*Gliricidia maculata* (Kunth) Steud.	Campeche, Quintana Roo, Yucatan	*
	*Havardia albicans* (Kunth) Britton & Rose	Campeche, Quintana Roo, Yucatan	*
	*Lonchocarpus castilloi* Standl.	Campeche, Quintana Roo, Yucatan	*
	*L. xuul* Lundell	Campeche, Quintana Roo, Yucatan	*
	*L. yucatanensis* Pittier	Campeche, Quintana Roo, Yucatan	*
	*Machaerium ramosiae* J. Linares	Campeche	**
	*Mariosousa dolichostachya* (S.F. Blake) Seingler & Ebinger	Campeche, Quintana Roo, Yucatan	*
	*Platymiscium yucatanum* Standl.	Campeche, Quintana Roo, Yucatan	*
	*Prosopis mayana* R. A Palacios	Yucatan	
	*Rhynchosia yucatanensis* Verde	Campeche, Yucatan	
	*Senegalia gaumeri* (S. F. Blake) Britton & Rose	Campeche, Quintana Roo, Yucatan	*
	*Senna pallida* (Vahl) H. S. Irwun & Barneby	Quintana Roo	
	*Stylosanthes quintanarooensis* Gama & Dávila	Quintana Roo	
Gentianaceae	*Lisianthius axillaris* (HemsI.) Kuntze	Campeche, Quintana Roo, Yucatan	*
Icacinaceae	*Ottoschulzia pallida* Lundell	Campeche, Quintana Roo	
Lamiaceae	*Hiptis* sp. Jacq.	Yucatan	
	*Salvia fernaldii* Standl.	Campeche, Quintana Roo, Yucatan	
Lythraceae	*Cuphea gaumeri* Koehne	Campeche, Quintana Roo, Yucatan	
Malpighiaceae	*Byrsonima bucidifolia* Standl.	Campeche, Quintana Roo, Yucatan	*
	*Malpighia souzae* Miranda	Campeche, Yucatan	
	*M. yucatanaea* F. K. Mey.	Campeche, Yucatan	
Malvaceae	*Ayenia fasciculata* Millsp. ex Standl.	Quintana Roo, Yucatan	
	*Bakeridesia yucatana* (Standl.) D. M. Bates	Quintana Roo	
	*Ceiba schottii* Britten & Baker f.	Campeche, Quintana Roo, Yucatan	*
	*Hampea trilobata* Standl.	Campeche, Quintana Roo	*
Melanthiaceae	*Schoenocaulon yucatanense* Brinker	Campeche, Quintana Roo, Yucatan	
Myrtaceae	*Calyptranthes karlingii* Standl.	Campeche, Quintana Roo	*
	*Eugenia bumelioides* Standl.	Yucatan	*
	*E. ibarrae* Lundell	Campeche	*
	*E. trikii* Lundell	Campeche, Quintana Roo	*
	*E. winzerlingii* Standl.	Campeche, Quintana Roo	*
	*Mosiera contrerasii* (Lundell) Landrum	Quintana Roo	*
	*Myrciaria ibarrae* de Lundell	Campeche, Quintana Roo	*
Nolinaceae	*Beaucarnea pliabilis* (Baker) Rose	Campeche, Quintana Roo, Yucatan	*
Orchidaceae	*Cohniella ascendens* (Lindl.) Christenson *x Lophiaris oerstedii* (Rchb.f.) R. Jiménez & Carnevali & Dressler	Quintana Roo	
	*Cohniella yucatanensis* Cetzal & Carnevali	Campeche, Quintana Roo, Yucatan	
	*Dendrophylax* sp. Rchb.f.	Yucatan	
	*Encyclia guatemalensis* (Klotzsch) Dressler & G. E. Pollard	Campeche, Quintana Roo, Yucatan	*
	*E. nematocaulon* (A. Rich.) Acuña *x Encyclia bractescens* (Lindl.) Hoehne	Yucatan	
	*Epidendrum martinezii* L. Sánchez & Carnevali	Quintana Roo	*
	*Habenaria leon-ibarrae* R. Jiménez & Carnevali	Quintana Roo	
	*Lophiaris andrewsiae* R. Jiménez & Carnevali	Campeche, Quintana Roo, Yucatan	
	*L. lindenii* (Brongn.) Braem *x Lophiaris oerstedii* (Rchb.f.) R. Jiménez & Carnevali	Yucatan	
	*L. tapiae* (Balam & Carnevali) J. M. H. Shaw	Campeche	**
	*Maxillariella yucatanensis* Carnevali & R. Jiménez	Campeche, Quintana Roo	
	*Myrmecophila christinae var. cristinae* Carnevali & Gómez-Juárez	Campeche, Quintana Roo, Yucatan	*
	*M. christinae var. ibarrae* Carnevali & J. L. Tapia	Campeche, Quintana Roo	
	*M. laguna-guerrerae* Carnevali, L. Ibarra & J. L. Tapia	Quintana Roo	
	*Ponthieva parviflora* Ames & C. Schweinf	Campeche, Quintana Roo	
	*Rhyncholaelia digbyana var. digbyana* (Lindl.) Schltr.	Campeche, Quintana Roo, Yucatan	
	*Trichosalpinx* sp. Luer	Quintana Roo	
Passifloraceae	*Passiflora itzensis* (J. M. MacDougal) Puerto-Utl.	Quintana Roo, Yucatan	*
	*P. mayarum* J. M. Macdougal	Campeche, Quintana Roo	*
	*P. sublanceolata* (Killip) J. M. MacDougal	Campeche, Quintana Roo, Yucatan	*
	*P. urbaniana* Killip	Campeche	*
	*P. xiikzodz* J. M. MacDougal	Campeche, Quintana Roo. Yucatan	*
	*P. yucatanensis* Killip ex Standl.	Campeche, Quintana Roo	
Piperaceaae	*Piper cordoncillo var. apazoteanum* Trel.	Campeche	**
Plantaginaceae	*Angelonia ciliaris* B. L. Rob.	Campeche, Quintana Roo, Yucatan	*
	*A. parviflora* Barringer	Campeche, Quintana Roo, Yucatan	
Poaceae	*Paspalum mayanum* Chase	Yucatan	
	*P. sparsum* Chase	Campeche, Yucatan	
	*Schizachyrium gaumeri* de Nash	Campeche, Yucatan	
	*Setaria variifolia* (Swallen) Davidse	Campeche, Quintana Roo, Yucatan	*
Polygonaceae	*Coccoloba ortizii* R. A. Howard	Quintana Roo, Yucatan	
	*Neomillspaughia emarginata* (H.Gross) S. F. Blake	Campeche, Quintana Roo, Yucatan	*
Pomeloniaceae	*Loeselia campechiana* C. Gut. Báez & Duno	Campeche	**
Primulaceae	*Bonellia albiflora* (Lundell) B.Ståhl & Källersjö	Campeche, Quintana Roo, Yucatan	*
	*B. flammea* (Millsp. ex Mez) B.Ståhl & Källersjö	Campeche, Quintana Roo, Yucatan	
	*B. sak-lol* Carnevali & J. L. Tapia	Quintana Roo	
Rhamnaceae	*Colubrina gregii var. yucatanensis* M. C. Johnst.	Campeche, Quintana Roo, Yucatan	*
	*Ziziphus yucatanensis* Standl.	Campeche, Quintana Roo, Yucatan	
Rubiaceae	*Alseis yucatanensis* Standl.	Campeche, Quintana Roo, Yucatan	*
	*Asemnantha pubescens* Hook. f.	Campeche, Quintana Roo, Yucatan	*
	*Cosmocalyx spectabilis* Standl.	Campeche, Quintana Roo, Yucatan	*
	*Guettarda gaumeri* Standl.	Campeche, Quintana Roo, Yucatan	*
	*Hintonia octomera* (Hemsl.) Bullock	Campeche, Quintana Roo, Yucatan	*
	*Machaonia lindeniana* Baill.	Campeche, Quintana Roo, Yucatan	*
	*Randia longiloba* Hemsl.	Campeche, Quintana Roo, Yucatan	
	*R. truncata* Greenm. & C. H. Homps.	Campeche, Quintana Roo, Yucatan	
	*Sabicea flagenioides* Wernham	Quintana Roo	
Rutaceae	*Pilocarpus racemosus var. yucatanus* Kaastra	Yucatan	
Salicaceae	*Casearia subsessiliflora* Lundell	Quintana Roo, Yucatan	
	*Samyda yucatanensis* Standl.	Campeche, Quintana Roo, Yucatan	
Santalaceae	*Phoradendron tikalense* Kuijt	Quintana Roo	*
Sapindaceae	*Serjania pterarthra* Standl.	Campeche, Quintana Roo, Yucatan	*
	*S. yucatanensis* Standl.	Campeche, Quintana Roo, Yucatan	*
	*Talisia floresii* Standl.	Campeche, Quintana Roo, Yucatan	*
	*Thouinia paucidentata* Radlk.	Campeche, Quintana Roo, Yucatan	*
Sapotaceae	*Sideroxylon foetidissimum* subsp. *gaumeri* Jacq.	Campeche, Quintana Roo, Yucatan	*
Verbenaceae	*Citharexylum calvum* Moldenke	Quintana Roo, Yucatan	
	*Lantana velutina* M. Martens & Galeotti	Campeche	**
	*Lippia yucatana* Loes.	Quintana Roo, Yucatan	
	*Stachytarpheta grisea* Moldenke	Campeche, Quintana Roo	
	*S. lundellae* Moldenke	Quintana Roo, Yucatan	
Violaceae	*Hybanthis mexicanus* subsp. *pilosus* H. E. Ballard & Wahlert	Yucatan	
n = 48	Genera: 135, Species: 201	123	78

* Present in the biotic province of the Yucatan Peninsula (including Central American countries). ** Endemic to the state of Campeche (not found elsewhere).

**Table 2:** Number of botanical genera and species present in the state of Campeche, belonging to the Yucatan Peninsula Biotic Province (YPBP) that have been studied for pests’ control.

Family	Occurrence		Previous Reports	
	Genera	Species	Genera	Species
Acanthaceae	3	6		
Agavaceae	1	1	1	1
Anacardiaceae	1	1		
Anthericaceae	1	2		
Apocynaceae	3	8		
Arecaceae	1	1		
Asteraceae	8	8		
Brassicaceae	1	1		
Bromeliaceae	2	3		
Cactaceae	2	3	1	1
Campanulaceae	1	1		
Capparaceae	1	3		
Celastraceae	1	1		
Convolvulaceae	1	2		
Cyperaceae	1	1	-	-
Dioscoreaceae	1	1	-	-
Ebenaceae	1	2	1	1
Erythroxylaceae	1	1	-	-
Euphorbiaceae	8	15	4	5
Fabaceae	12	15	5	7
Gentianaceae	1	1	-	-
Icacinaceae	1	1	-	-
Lamiaceae	1	1	-	-
Lythraceae	1	1	-	-
Malpighiaceae	2	3	-	-
Malvaceae	2	2	-	-
Melanthiaceae	1	1	-	-
Myrtaceae	3	5	1	1
Nolinaceae	1	1	-	-
Orchidaceae	7	9	-	-
Passifloraceae	1	5	-	-
Piperaceaae	1	1	-	-
Plantaginaceae	1	2	-	-
Poaceae	3	3	-	-
Polygonaceae	1	1	-	-
Pomeloniaceae	1	1	-	-
Primulaceae	1	2	1	2
Rhamnaceae	2	2	1	1
Rubiaceae	7	8	1	1
Salicaceae	1	1	1	1
Sapindaceae	3	4	1	1
Sapotaceae	1	1	1	1
Verbenaceae	2	2	-	-
n = 43	96	134	19	23

**Table 3:** Updated list of endemic plants of the Biotic Province of the Yucatan Peninsula (YPBP), with previous reports on chemical characterization and use for pest control.

Family	Plant Species	Target Pest ^1^	Botanical Extract	Dose of Botanical Extract Used ^2^	**Chemical Compounds Reported**	**Reference**
Agavaceae	*Furcraea cahum* Trel.	*Meloidogyne incognita* (Kofoid & White) Chitwood (Heteroderidae)	Ethanolic extract	500 and 250 ppm		[44]
		*Colletotrichum gloeosporioides* (Penz.) Penz. & Sacc. (Glomerellaceae)	Ethanolic extract	2 mg mL^−1^		[45]
		*Alternaria chrysanthemi* Simmons & Crosier (Pleosporaceae)	Ethanolic and aqueous extract	1 mg mL^−1^ and 3% *w*/*v*		[46]
Cactaceae	*Selenicereus grandiflorus* subsp. *donkelaarii* (Salm-Dyck) Ralf Bauer				Lupeol, Oleanolic acid, Betulinic acid	[47]
Ebenaceae	*Diospyros bumelioides* Standl.	*Staphylococcus aureus* Rosenbach (Staphylococcaceae) *Candida albicans* (C.P.Robin) Berkhout (Saccharomycetaceae) *Bacillus subtilis* (Ehrenberg) Cohn (Bacillaceae)	Methanolic bark and root extracts	<0.2 mg mL^−1^		[48]
Euphorbiaceae	*Acalypha gaumeri* Pax & K. Hoffm.	*M. incognita* (Kofoid & White) Chitwood (Heteroderidae)	Ethanolic crude extract	500 and 250 ppm		[44]
		*Fusarium oxysporum* Schltdl. (Nectriaceae) *Rhizopus* sp. Ehrenb (Mucoraceae)	Ethanolic crude extract	2 mg mL^−1^		[45]
		*A. chrysanthemi* Simmons & Crosier (Pleosporaceae)	Ethanolic and aqueous extract	IC_50_ = 0.53 mg mL^−1^		[46]
	*Cnidoscolus souzae* McVaugh	*Saccharomyces cerevisiae* Meyen ex E.C.Hansen (Saccharomycetaceae) *Candida albicans* (C.P.Robin) Berkhout (Saccharomycetaceae) *Aspergillus niger* P.E.L. van Tieghem (Trichocomaceae) *Trichophyton mentagrophytes* Robin (Arthrodermataceae)	Methanolic extract	1000 y 500 µg mL^−1^		[49]
		*Leishmania mexicana* Biagi, Emend, Garnham (Trypanosomatidae)	Methanolic extract	1, 10, 100 µg mL^−1^		[49]
			n-hexane fraction		Diterpenes	[50]
			Ethanolic extract		7-deoxynimbidiol	[51]
	*Croton arboreus* Millsp.		n-hexaneacetone extract		Sesquiterpenes	[52]
	*Croton chichenensis* Lundell	*M. incognita* (Kofoid & White) Chitwood (Heteroderidae)	Ethanolic crude extract	500 and 250 ppm		[44]
	*C. chichenensis*	*Alternaria tagetica* S. K. Shome & Mustafee (Pleosporaceae)	Ethanolic crude extract	2 mg mL^−1^		[45]
	*C. chichenensis*	*C. gloeosporioides* (Penz.) Penz. & Sacc. (Glomerellaceae)	Ethanolic and aqueous extract	MIC = ≤500–1000 μg mL^−1^)		[53]
	*C. chichenensis*	*A. chrysanthemi* Simmons & Crosier (Pleosporaceae)	Ethanolic and aqueous extract	IC_50_ = 0.53 mg mL^−1^		[46]
	*Jatropha gaumeri* Greenm.	*B. subtilis* Ehrenberg) Cohn (Bacillaceae)	Methanolic extract	25 μg	Jatrogrossidione	[54]
Fabaceae	*Gliricidia maculate* (Kunth) Steud.	*Paracoccus marginatus* Williams & Granara de Willink (Pseudococcidae) *Pseudococcus cryptus* Hempel (Pseudococcidae) *Planococcus citri* Risso (Pseudococcidae) *Planococcus minor* (Maskell) (Pseudococcidae)	Methanolic and aqueous extract	0.00–0.20%		[55]
	*Havardia albicans* (Kunth) Britton & Rose	*Rhipicephalus microplus* (Canestrini, Ixodidae, *larvae and adults)*	Methanolic extract	LC_50_ = 7.0%		[56]
		*Haemonchus contortus* (Rudolphi, Trichostrongyloidea)	Acetone-water extract (70:30)	600, 1200, 1800 and 2400 µg mL^−1^		[57]
	*Lonchocarpus castilloi* Standl.	*Cryptotermes brevis* (Walker, Kalotermitidae)	Hexane, acetone, methanol, water extract	2.5% *w*/*v*	Castillen D and Castillen E	[58]
		*Lenzites trabea* Fr. (Pers.) (Gloeophyllaceae)	Pure compound	0.25 mg mL^−1^	Castillene	[59]
	*Lonchocarpus xuul* Lundell	*Trypanosoma cruzi* Chagas (Trypanosomatidae) *Leishmania braziliensis* Vianna (Trypanosomatidae) *Leishmania amazonensis* Lainson & Shaw (Trypanosomatidae) *Leishmania donovani* (Laveran & Mesnil) Ross (Trypanosomatidae)	Pure flavonoids	50, 20, 10, 1 and 0.1 mg mL^−1^	Flavonoids, chalcone 7, flavan 3 and flavone 6	[60]
	*Lonchocarpus yucatanensis* Pittier		n-hexane and hexane: ethyl acetate mixtures		Flavonoids	[61]
			n-hexane-acetone mixtures		dihydroisocordoin and phlemistrictin	[62]
	*Platymiscium yucatanum* Standl.	*L. mexicana* Biagi, Emend, Garnham (Trypanosomatidae)	Methanolic extract	IC_50_ = 2.56 μg mL^−1^		[49]
	*Senegalia gaumeri* (S. F. Blake) Britton & Rose	*Haemonchus contortus* (Rudolphi) Cobb (Trichostrongyloidea, *egss and larvae)*	Methanol-water extract	80.29%, LC = 58.9 μgmL^−1^	fatty acids	[63]
Myrtaceae	*Eugenia winzerlingii* Standl.	*M. incognita* (Kofoid & White) Chitwood (Heteroderidae)	Ethanolic crude extract	500 and 250 ppm		[44]
		*A. tagetica* S. K. Shome & Mustafee (Pleosporaceae) *Rhizopus* sp. Ehrenb (Mucoraceae)	Ethanolic crude extract	2 mg mL^−1^		[45]
		*Bemisia tabaci* (Gennadius, Aleyrodidae, eggs, nymphs, and adults)	Aqueous and ethyl acetate extract	LC_50_ = 0.21 y 1.29% *w*/*v* (eggs)		[64]
Primulaceae	*Bonellia albiflora* (Lundell) B. Ståhl & Källersjö	*L. mexicana* Biagi, Emend, Garnham (Trypanosomatidae)	Methanolic extract	IC_50_ = 2.56 μg mL^−1^		[49]
	*Bonellia flammea* (Millsp. ex Mez) B. Ståhl & Källersjö	*Curvularia verruculosa* Tandon & Bilgrami ex M.B.Ellis (Pleosporaceae) *Curvularia lunata* (Wakker) Boedijn (Pleosporaceae) *Exserohilum rostratum* (Drechsler) K.J.Leonard & Suggs (Pleosporaceae) *Bipolaris setariae* Shoemaker (Pleosporaceae) *Corynespora cassiicola* (Berk. & M. A.Curtis) C. T. Wei (Corynesporascaceae) *Lasiodiplodia parva* A. J. L. Phillips, A. Alves & Crous (Botryosphaeriaceae)	Aqueous extract	3% (30 g L^−1^)		[65]
Rhamnaceae	*Colubrina gregii var. yucatanensis* M. C. Johnst.	*T. cruzi* Chagas (Trypanosomatidae) *L. amazonensis* ainson & Shaw (Trypanosomatidae)	Ethanolic crude extract	≤100 µg mL^−1^	Triterpenes	[66]
		*Candida albicans* C.P.Robin) Berkhout (Saccharomycetaceae) *C. glabrata* (H. W. Anderson) S. A. Mey. & Yarrow (Saccharomycetaceae) *C. parapsilosis* (Ashford) Langeron & Talice (Saccharomycetaceae) *C. krusei* (Castell.) Berkhout (Saccharomycetaceae) *C. tropicalis* (Castell.) Berkhout (Saccharomycetaceae)	Ethyl acetate and butanol extract	125 μg mL^−1^	Flavonoids	[67]
Rubiaceae	*Randia longiloba* Hemsl.	*M. incognita* (Kofoid & White) Chitwood (Heteroderidae)	Ethanolic crude extract	500 and 250 ppm		[44]
		*Rhizopus* sp. Ehrenb (Mucoraceae)	Ethanolic crude extract	2 mg mL^−1^		[45]
Salicaceae	*Samyda yucatanensis* Standl.	*Atta mexicana* (Smith, Formicidae)	Ethanolic extract	40 mg cm^−1^	Sesquiterpene farnesol	[68]
Sapindaceae	*Serjania yucatanensis* Standl.	*T. cruzi* Chagas (Trypanosomatidae)	Ethanolic extract	100, 50 and 25 µg mL^−1^		[69]
Sapotaceae	*Sideroxylon foetidissimum* subsp. *gaumeri* (Pittier) T. D. Penn.		Ethanolic extract		Triterpene, Saponin	[70]

^1^ A pest is any organism that, when interacting with human systems, causes economic, environmental or social damage. This term includes insects, weeds, bacteria, fungi, viruses, vertebrates and other living organisms that interfere with human activities [1]; ^2^ Specific amount of plant extract applied to control a pest. IC_50_: Mean Inhibitory Concentration; MIC: Minimum inhibitory concentration; LC_50_: Mean lethal concentration.

## Data Availability

All the associated data are available in the manuscript.
